# Exploring good mental health for people with intellectual disabilities: a qualitative interview study with mental health experts

**DOI:** 10.1186/s12939-025-02540-0

**Published:** 2025-06-12

**Authors:** Sophie Komenda-Schned, Paula Moritz, Sarah Jasmin Landskron, Alma Rosa Herscovici, Charlotte Schomburg, Julia Lehner, Brigitte Lueger-Schuster, Luis Salvador-Carulla, Elisabeth Lucia Zeilinger

**Affiliations:** 1 Department of Clinical and Health Psychology, Faculty of Psychology, University of Vienna, Liebiggasse 5, Vienna, A-1010 Austria; 2 Vienna Doctoral School in Cognition, Behavior and Neuroscience, University of Vienna, Vienna, Austria; 3 Research Institute for Developmental Medicine, Johannes Kepler University Linz, Linz, Austria; 4 Health Research Institute, Faculty of Health, University of Canberra, ACT, Canberra, Australia; 5 Department of Clinical Research SBG, Academy for Ageing Research, Haus der Barmherzigkeit, Vienna, Austria

**Keywords:** Intellectual disabilities, Good mental health, Expert interviews, Psychosocial functioning, Reflexive thematic analysis

## Abstract

**Background:**

Most mental health research focuses on mental disorders, with a paucity of studies exploring good mental health. Widely used definitions of good mental health (e.g., WHO’s) include several aspects of positive human functioning as predisposing to mental health. According to diagnostic criteria, people with intellectual disabilities (ID) are impaired in their functioning levels (e.g., intellectual functioning, adaptive skills). Existing definitions of good mental health may therefore not be applicable to an ID population.

**Objective:**

We aimed to explore the perspectives of experts on good mental health in people with ID to shed light on the constitutive factors of good mental health in this population.

**Methods:**

Semi-structured expert interviews with open-ended questions (*N* = 12) were conducted with psychiatrists, psychologists, psychotherapists, and psychiatric nurses, working either academically or in practice with this population. Inductive, reflexive thematic analysis was applied to analyze and synthesize the data.

**Results:**

Four themes regarding components of good mental health in people with ID were derived: (1) Absence of Mental Illness, (2) Physical Health, (3) Psychosocial Functioning, and (4) Social Environment. Psychosocial functioning was divided into five subthemes, namely: (1) Emotional Competence, (2) Self-Concept, (3) Experience of Meaning, (4) Self-Determination, and (5) Personal Growth.

**Conclusions:**

According the participants of this study, the constitutive factors of good mental health are similar for people with and without ID. The importance of needs-based, individualized support and an appropriate social environment was particularly emphasized for people with ID, to facilitate positive human functioning and promote good mental health.

**Supplementary Information:**

The online version contains supplementary material available at 10.1186/s12939-025-02540-0.

## Accessible Summary

• There is too little research on good mental health of people with learning disabilities. (The term learning disabilities is widely used by people with intellectual disabilities to refer to their own group [1]).

• Good mental health might be different for people with and without learning disabilities We want to find out which factors contribute to good mental health of people with learning disabilities.

• We interviewed 12 mental health experts and we summarized their responses.

• Important aspects of good mental health are:

1. Not having mental illness (e.g., no depression).

2. Physical health (e.g., eating healthy, doing enough sports).

3. Psychosocial aspects (e.g., dealing with emotions, doing something meaningful, making my own decisions).

4. Social network and support environment (e.g., having close friends, getting the support I need).

• Some mental health experts said that facets of good mental health are similar for people with and without learning disabilities They also said that getting the right support and having the right people around you is very important for people with learning disabilities.

## Introduction

Intellectual disabilities (ID) originate early in life and are characterized by reduced intellectual functioning and adaptive behavior [2]. Mild, moderate, severe, and profound forms of ID can be distinguished. In the latest releases of the ICD-11 and the DSM-5, the diagnostic criteria shifted from relying mostly on IQ-measures to focusing on behavioral indicators of adaptive functioning, thereby allowing a more comprehensive description of a person’s needs and abilities [2, 3].

While the ICD-11 [2] and DSM-5 [3] focus on medical classification, *The International Classification of Functioning*, *Disability and Health* (ICF) [4] complements this by offering more holistic and individualistic descriptions of people with ID. The ICF is a widely used tool to describe health and functioning as interacting bodily functions and structures, activities, participation, environmental factors, and personal factors. Based on the ICF, Luckasson and Schalock [5] developed the functionality model of ID, which proposes an integrated system: (1) human functioning dimensions (intellectual functioning, adaptive skills, health, participation, and personal and environmental factors) act as input component, (2) systems of supports (support services, accommodation, technology, policies, and practice) as throughput, and (3) human functioning outcomes (socio-economic status, health status, and well-being) as output component [5]. People with ID experience limitations in human functioning dimensions and are likely to depend on support in different areas of their daily life. According to the ICF [4] and the functionality model [5], this puts them at risk for reduced functioning outcomes, including their mental health status [6].

Like any other person, people with ID can experience good mental health as well as mental health problems. From a lay perspective mental health is frequently described as a unidimensional continuum from healthy to ill. However, this continuum approach fails to display the complexity of the construct. In the current scientific discourse, mental health and mental illness are better described as correlated, but distinct [7]. In case of mental illness in people with ID, the term ‘double diagnosis’ is frequently used, as they not only have an ID-diagnosis, but also an additional psychiatric disorder (i.e., comorbidities). However, psychiatric disorders are difficult to diagnose in people with ID. Diagnostic overshadowing (i.e., seeing symptoms as part of the ID rather than a mental disorder) [8, 9], and the fact that symptoms may present differently in this population, frequently hinder early and adequate diagnosis [6, 10]. The prevalence rates of mental health problems are increased in people with ID [6] compared to people without ID due to greater exposure to risk factors. These include trauma, low socioeconomic status, reduced physical and cognitive functioning, limitations in coping strategies or social skills [10–12]. Moreover, there is a lack of specialized and appropriate strategies to promote good mental health and prevent psychiatric disorders in this population [13, 14].

For the general population, good mental health is most prominently defined by the World Health Organization (WHO) as “a state of well-being in which every individual realizes his or her own potential, can cope with the normal stresses of life, can work productively and fruitfully, and is able to make a contribution to her or his community” [15]. More recently, Galderisi and colleagues [16] proposed that mental health is more than just wellbeing, positive emotions and positive functioning. They defined good mental health as:

*A dynamic state of internal equilibrium which enables individuals to use their abilities in harmony with universal values of society. Basic cognitive and social skills; ability to recognize*, *express and modulate one’s own emotions*, *as well as empathize with others; flexibility and ability to cope with adverse life events and function in social roles; and harmonious relationship between body and mind represent important components of mental health which contribute*, *to varying degrees*, *to the state of internal equilibrium* [16]. ^(p. 231–232)^

However, when applied to people with ID both definitions share similar difficulties: they encompass multiple aspects of functioning dimensions, like cognitive functioning or adaptive abilities [5], which are limited in people with ID due to their disability. This raises questions about the suitability of these definitions for people with ID and underscores the need for a more tailored understanding of mental health within this group.

Addressing this gap is particularly important given the mandate for high-quality healthcare for people with ID outlined in the Convention on the Rights of Persons with Disabilities by the United Nations (UN-CRPD) [17]. Article 25 of the UN-CRPD entitles people with ID to receive the “highest attainable standard of health without discrimination on the basis of disability” [17]. This includes “the same range, quality and standard of […] healthcare and […] population-based public health programs” [17]. Currently, there is insufficient evidence on theories, concepts, and definitions of good mental health specifically for people with ID [18–21], which limits the development of effective mental health promotion and prevention programs tailored to this population. A recent systematic literature review found no studies evaluating the suitability of existing definitions of good mental health for people with ID, nor any adapted definitions designed for this group [20]. While there is a substantial body of research on related constructs such as wellbeing, quality of life, and positive psychology constructs [7, 20, 22], as well as on mental disorders [6, 23–25], a clear and agreed-upon definition of good mental health for people with ID is notably absent. This indicated not only a violation of the UN-CRPD [17], but also highlights a major research gap. The development of a clear definition of good mental health for people with ID is essential to provide a strong evidence base for mental health promotion and prevention programs that address their needs and take into account their increased risk of developing mental disorders [6, 26, 27].

We aim to address this research gap within a larger multi-method research project that encompasses focus groups with people with ID, a systematic review on good mental health in people with ID, which was only able to identify good mental health definitions developed for the general population [20], and semi-structured interviews with international experts in mental health for people with ID. Expert interviews are a promising approach to gather novel aspects and generate new knowledge on previously under-researched topics [28, 29], as experts can provide valuable insights into their professional experiences in scientific and clinical settings [30]. The present study therefore aimed to explore expert’s perspectives on constitutive and contributing factors to good mental health of people with ID.

## Materials and methods

### Measures

The interview questions were developed by two psychologists (SKS, PM) with scientific and practical experience in the field of mental health and ID. As they were also part of the research team conducting a systematic review on the topic [20] special consideration was taken not to confound the interview guidelines with any a priori assumptions. This risk was, however, relatively low since the aim of this study was not to confirm or disconfirm the reviews results, but to explore expert’s perspectives. According to the approach of Helfferich [31] the design of the semi-structured interview guidelines was not theory-driven, but rather explorative. All questions were formulated in an open-ended manner to avoid bias from a priori assumptions and to allow experts to freely elaborate on their positions. The interview questions addressed the following topics: (1) good mental health in the general population vs. in an ID population, (2) constitutive factors of good mental health for people with ID, (3) possible differences in good mental health of people with different levels of functioning or ID-levels, and (4) mental health promotion in people with ID. After the interview, participants were invited to identify the most important factors of good mental health in people with ID, based on their own elaborations within the interview, with a simple, open-ended question. The interview guidelines are provided in Supplementary File A.

### Procedure and sampling strategy

Participants had to meet the following inclusion criteria as experts based on the concept of Gläser and Laudel [32]: (1) mental health professionals: psychiatrists, psychologists/psychotherapists, psychiatric nurses working either practically and/or scientifically with people with ID, (2) knowledge and several years of experience in the field of good mental health, mental health promotion and ID, (3) English or German language skills to conduct the interview. We aimed to recruit a maximum variation sample, encompassing both in- and outpatient units, as well as scientific institutions. Additionally, diversity in terms of age, gender, nationality, profession, and experience was intended.

Recruitment of participants was guided by the e-mail lists of national and European organizations that address the work for and with people with ID, such as the European Association for Mental Health in Intellectual Disability (EAMHID), or the ID-Group of the Austrian Association for Psychiatry, Psychotherapy, and Psychosomatics (ÖGPP). In sum, 22 national and European experts with clinical experience working with people with ID were purposefully selected based on their professions, work settings, experience, age, gender, etc. and contacted via e-mail. An overview of their demographics is displayed in Supplementary File B. The invitation letter was appended to the recruitment emails. It provided an overview of the study topic and design, as well as contact details of the researchers. Information about the study topic was kept short, no information about existing definitions or conceptualizations of good mental health and their possible difficulties in an ID population were included in the invitation letter to avoid biasing potential participant’s views. No incentives were provided to encourage participation.

A total of 12 interviews were conducted from April 2023 to June 2023. Following the electronic signature of the informed consent document, demographic information was collected using a web-based survey tool. Interviews were conducted by two trained members of the research team (SKS, PM). Ten interviews were conducted online, contingent on the location and preference of the interviewee. The duration of the interviews ranged from 39 to 66 min (*M* = 47 min). Audio recordings were made, and notes were taken. A verbatim transcription was produced using the audio and the written notes. The software TRINT [33] was used to facilitate transcription. Ethical approval for the study was obtained from the Ethics Committee of the study site (No. 00885, date of approval: November 25th, 2022).

### Data analysis

To analyze interview data, inductive, reflexive thematic analysis was employed [34]. We applied a relativist, experimental, and semantic approach, whereby the analysis is guided by the data itself rather than by theoretically derived preconceptions, focusing solely on the participants’ perspectives. MAXQDA v.2022 [35] was utilized to aid data analysis. Three out of four members of the coding team were naïve to the systematic review [20] and its results and coding took place collectively or in pairs to prevent bias from prior analysis. Initially, a set of three interviews with representatives of different mental health professions, reporting a broad range of experts’ perspectives, was collectively coded by all members of the coding team (PM, AH, JL, CS) to develop a comprehensive and consistent coding scheme. The remaining interviews were then coded by pairs of researchers to prevent subjective bias, with novel codes being discussed within the coding team. Afterwards, new codes were applied to all interviews. However, new codes only marginally augmented the coding scheme on the level of single codes added to subthemes. Coding of the final interview did not require any further changes to the coding scheme, indicating data redundancy and code saturation [36, 37]. The coding team collaboratively clustered the final codes, identified themes, and examined their hierarchies. To avoid interpretation bias, two researchers who were not previously involved in the coding process (SKS, SJL) reviewed and adapted the thematic mapping. Finally, the original coding team revised the analytical themes to ensure fit to the data.

The final interview question, asking for the most important factors of good mental health, was analyzed differently. The important factors mentioned by the experts were summarized and clustered to themes. Thereafter, the frequencies of the themes were counted, and clusters with four or more nominations were reported.

## Results

### Participants

The final sample comprised 12 experts from three European countries (Austria, Germany, Netherlands). Participants identified as female (9), male (2), and diverse (1). Five psychiatrists, four psychologists/psychotherapists, and three psychiatric nurses took part in this study. Participant age ranged from 30 to 73 years (*M* = 51.5, *SD* = 11.8). On average, they had 22.1 years of experience in their profession (*SD* = 1.7, range: 4.5–48.0). Participation rate for this study was 54.5%, i.e., 12 out of 22 contacted experts agreed to participate.

### Main themes derived from the data

**Fig. 1:**
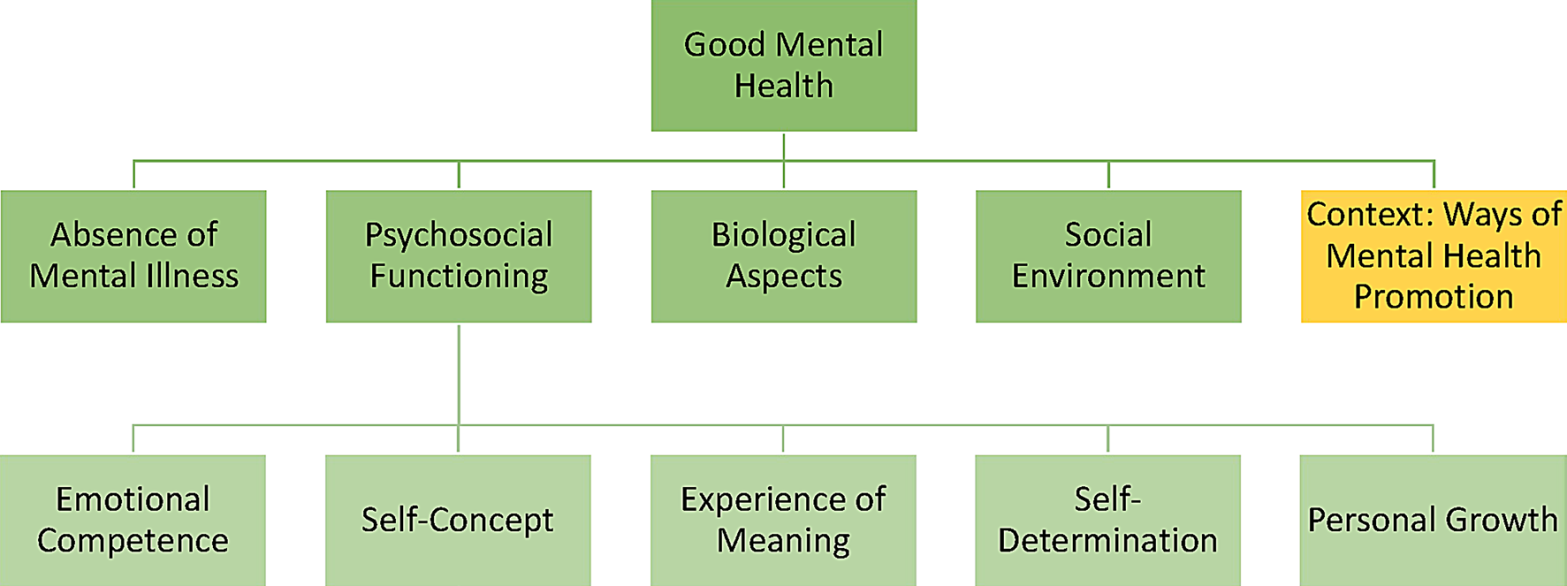
Themes and subthemes of the qualitative analysis The figure shows the themes and subthemes derived from the qualitative analysis of the twelve expert interviews

Four themes regarding components of good mental health in people with ID were derived from the interview data: (1) Absence of Mental Illness, (2) Physical Health, (3) Psychosocial Functioning, and (4) Social Environment. Psychosocial Functioning was divided into five subthemes. Additionally, Ways of Mental Health Promotion was identified as a contextual theme and is presented in Supplementary File C. Figure 1 provides an overview of all themes and subthemes.

Fig. 1 should be placed here -Participants reported that the construct of good mental health was hard to define and lacked tangibility. They agreed that good mental health is highly subjective and that intra-individual variabilities, such as behavior or mood, should be considered. The manifestation of good mental health may vary according to the individual’s level of functioning, for example, in relation to the severity of ID or the independence in daily life. However, the experts’ opinions were discordant regarding the impact of functioning levels on good mental health. Irrespective of functioning levels, participants emphasized the role of an adequate person-environment fit and the provision of needs-based support, not only on a practical, but also on a socio-emotional level, thereby promoting participation and self-determination. Almost all participants concurred that there is no or only a little difference regarding mental health of people with and without ID. The participants defined good mental health as being even tempered, feeling well, and having a good quality of life.

### Absence of mental illness

In most of the interviews, as a first association, mental health was initially equated with mental illness, challenging behavior or deviation of personal norm behavior. After debriefing, good mental health was often described by the absence of mental illness: ‘*Mental health is the absence of [mental] illness. And from a medical perspective*, *this is clearly defined by certain syndromes*, *which are described in the ICD-11 or the DSM-5’* (Interview 3). In particular, the absence of specific symptoms and symptom clusters, like psychotic symptoms, anxiety or obsessive-compulsive symptoms was emphasized by some of the experts.

### Physical health

Physical health was seen as influential for mental health by the experts. ‘*There is another dimension*, *that we always have to think about: the physical dimension. I’m assuming from a mental health perspective*, *that someone who has physical difficulties will naturally also has an impact on their mental health.*’ (Interview 7). In particular, the influences of (good) nutrition and movement were emphasized in this context.

### Psychosocial functioning

Psychosocial functioning was the richest theme in this thematic analysis. Based on its comprehensiveness, it was divided into five subthemes, namely: (1) Emotional Competence, (2) Self-Concept, (3) Experience of Meaning, (4) Self-Determination, (5) Personal Growth.

#### Emotional competence

Emotional competence was split into two categories: the perception of emotions and emotion regulation. While perception of emotions referred to recognizing one’s own emotions and being able to name or describe them, emotion regulation included dealing with one’s own emotions as well as impulse control. ‘*To have the feeling that you can deal with your emotions. That doesn’t mean*, *that you are always fine*, *but that you know what you can do with them [the emotions]. That you have strategies to get yourself out of holes. Yes*, *I think dealing with your own emotionality’* (Interview 4). Another mental health expert described emotion regulation in people with ID a more practical matter: ‘*It’s more about understanding emojis correctly*, *or not taking it personally*, *if somebody doesn’t reply to a WhatsApp message for a long time’* (Interview 3).

#### Self-concept

Self-concept included aspects of self-acceptance, self-confidence, and self-perception. ‘*Getting a feeling about what makes me who I am? How powerful am I? How can I relate well? What do others think about me?’* (Interview 10). Or ‘*a YES towards oneself*” (Interview 11). Likewise, being able to be oneself and knowing what is good for oneself, are part of this subtheme: ‘*I know what is good for me and what I need’* (Interview 9).

#### Experience of meaning

The subtheme experience of meaning was structured into the domains of competence, meaningful activities, and spirituality. The experience of competence encompasses perceiving one’s own strength and talents. Pursuing hobbies and interests can be subsumed under engaging in meaningful activities, as one expert explained ‘*I think [for people with ID] it´s the same things we do. Finding hobbies and interests. Anyone can do that. Things that you like doing*, *recognizing what is good for you and doing more of it.’* (Interview 7). Spirituality involves believing in something and is reflected in aspects like *’going to church activities’* (Interview 8). Overall, meaningful experiences are about ‘*[T]he sense of being useful in this world.’* (Interview 12).

#### Self-determination

Self-determination encompassed several aspects, including the contrast between self-determination and external determination (focusing on possibility), acting autonomously (focusing on action), and self-efficacy. These facets centered on ensuring ‘*that people [with ID] get a say [in] things*, *that they can make decisions*, *appropriate for what they can decide about.’* (Interview 12). This perspective was reinforced by the statement that ‘*The ability to contribute*, *to do something independently*, *is inherent in every person and is also seemingly programmed as a developmental goal. Of course*, *not everyone can do everything at every stage of [social*, *intellectual*, *and emotional] development. But independence appropriate to the stage of development is an important basic element.’* (Interview 11). Moreover, self-determined decision making is especially relevant in supported living facilities: *‘because when the weather is nice and I want to take a walk or meet up with friends. This would be easier if I lived in my own flat with no or little support. In a supported living facility*, *there are more rules*, *and I would have to ask a caregiver if I’m allowed to go out and I would have to explain what I plan to do and when I will be back home’* (Interview 7). Also, people with ID should be ‘*included in the decision about new roommates. Whether or not they thought the potential new roommate would be a good addition to the existing group. I experienced that only once in my career*, *but that was a great example for self-determination’* (Interview 9).

#### Personal growth

Personal growth could be described as unfolding, developing further, and exploring new experiences. Resilience takes a pivotal role in this process as one participant stated: ‘*It’s being resilient*, *happy and vital []*, *being curious and trying new things. In short*, *resilience*.*’* (Interview 6). Moreover, personal growth is shaped by personal experiences and events of one’s past, both positive and negative. As one interviewee stated: ‘*Generally speaking*, *I would define this [good mental health for people with ID] as being able to flourish depending on your developmental age (…) not influenced by internal nor external challenges.’* (Interview 10). Another essential aspect of personal growth is ‘*the opportunity for self-expression’* (Interview 2), especially in the context of their living environment ‘*[…] people with ID need a place. A safe place. […] a place where I can be who I am*, *where I am accepted as I am*, *and where I also have the opportunity to grow.’* (Interview 6).

### Social environment

Social environment was the second richest theme of this thematic analysis and was therefore divided into three subthemes: (1) Micro Level, (2) Meso Level, (3) Macro Level.

#### Micro level

The micro level included proximal factors of social environment, like sense of safety, and sense of belonging. For the mental health experts, it was important *‘[to] make sure*, *that people [with ID] feel connected. […] Make sure*, *that there is a sense of belonging and a place in this world’* (Interview 12). Likewise, emotional proximity, *‘Affection. Warmth. Love’* (Interview 2) and living out sexuality were named crucial for the closest level of social environment.

#### Meso level

The meso level of social environment mainly referred to the social network of people with ID, which can be dived into personal (e.g., family and friends) and professional relationships (e.g., caregivers, physicians, psychologists). Thereby, consistency and reliability were named as the most important factors: ‘*Constant relationships. I think that´s really important [for people with ID]*’ (Interview 6). Other mental health experts stressed ‘*to ensure that social relationships are offered that the person can accept as beneficial’* (Interview 11). In this context, being respected and ensuring mutual understanding were addressed. However, person-centered and needs-based support, e.g., ‘*Walking the tightrope between too much and too little support’* (Interview 8) or ‘*simply more INDIVIDUAL [support] concepts*’ (Interview 4) as well as a good person-environment-fit, which can be characterized by ‘*a healthy fitting living environment’* (Interview 12) *‘whichis not overchallenging*, *nor underchallenging’* (Interview 1), were discussed by the health experts.

#### Macro level

The macro level encompassed social issues such as the political environment, societal structures and mechanisms or the understanding of disabilities. The political environment refers to topics like accessibility, access to work or the living situation of people with ID. From the mental health experts´ point of view, it is important ‘*[to] insist more vehemently on their [people with ID´s] rights. Some people are already doing that*, *and I think we should do it even more’* (Interview 8). Another important aspect is promoting the social model, rather than adhering to the medical model of disability: *‘Letting people with disabilities live as they are. And to adapt the environment to THEM and not the other way around. […] In other words*, *they [people with ID] don´t have to adapt to the way things are*, *but the environment adapts to THEM’* (Interview 2). *‘Participation in social life’* (Interview 3) instead of exclusion, stigmatization, or the society´s lack of awareness of people with ID´s needs, were addressed as crucial for mental health in this population.

### Prioritizing the most important aspects of good mental health

When asked for the most important factors for good mental health of people with ID the interviewees concurringly prioritized (1) ‘*having a benevolent and supportive environment that cares*’ (Interview 7) and (2) ’*self-determination*’ (Interview 9) in equal measure.

This was followed by (3) respect for people with ID, which shows in ‘*treating [people with ID] as adults*’ (Interview 8) and ‘*having a different perspective on people with ID that doesn´t just see them in terms of their shortcomings*’ (Interview 6). Likewise, (4) social participation played a crucial role for the mental health experts, as one interviewee explained ‘*people with ID should of course be part of the community*’ (Interview 1).

## Discussion

This study offers first insights into the perspectives of experts regarding the concept of good mental health in people with ID. The following factors were identified: (1) Absence of Mental Illness, (2) Physical Health, (3) Psychosocial Functioning, and (4) Social Environment. The theme Psychosocial Functioning consists of five subthemes: (1) Emotional Competence, (2) Self Concept, (3) Experience of Meaning, (4) Self-Determination, and (5) Personal Growth. Participants initially equated mental health with (the absence of) mental disorders, a common misconception known from previous literature [38]. However, after debriefing positive aspects of good mental health, such as being even-tempered or feeling well, far outweighed negative ones.

The findings of this study include many aspects of good mental health mentioned in the WHO definition of good mental health [15] and the definition by Galderisi and colleagues [16]. However, basic cognitive skills, working productively and fruitfully, and functioning in different social roles were not mentioned to be important for good mental health of people with ID by the participants. The experts agreed that for people with ID their own perceived competence and their experience of meaning, such as activities, hobbies, or personal interests, is of greater value than their productivity at a (sheltered) workplace. The importance of flourishing, personal development and growth was emphasized. Moreover, in terms of social environment, our findings go beyond the facets of good mental health mentioned in both definitions: The experts stressed the importance of consistent and reliable, positive social relationships on the micro and meso level as well as the experience of inclusion in the community on the macro level. This is consistent with the findings of research conducted on the prevention of challenging behavior, which has been found to serve as an indicator of mental ill health [39].

In line with previous studies [40–42], an appropriate person-environment-fit was highlighted, implying neither over- nor under-challenging a person with ID and providing needs-based, person-centered support. In this regard, the relevance of appropriate housing for people with ID (e.g., private housing with or without support/family members, supported living facilities) that promotes a person’s self-determination and personal growth was emphasized.

The factors mentioned by the experts are highly concurrent to the findings of our previously conducted systematic literature review [20]. Even though some of the (sub-)themes were named differently, their content broadly overlapped, with variations in focus and depth. Some aspects, however, could only be found in the review’s results, like productivity at work or education, optimism, and a positive overall appraisal of life. Also, certain aspects of physical health, like health behaviors, vitality or the absence of physical pain, were more prominent within the systematic review’s results [20]. The interview’s results, however, go beyond the review’s results and provide deeper insights into the supportive nature of social relationships for people with ID. The importance of consistency and reliability in personal as well as professional relationships was particularly stressed. Moreover, the significance of personal growth was discussed in much more detail within the interviews. People with ID should be provided with opportunities to develop further and gain new experiences. Interviewees linked that to flourishing and resilience.

Our findings provide significant support for the existing frameworks of functioning, particularly the ICF [4], as well as the model by Luckasson and Schalock [5]. Mental health experts highlighted the importance of human functioning dimensions, such as adaptive behavior or participation, and optimized systems of support, such as support services or accommodation, for achieving good mental health outcomes. However, regarding levels of intellectual functioning – another functioning dimension – the experts’ opinions were discordant as to whether higher or lower levels of intellectual functioning impact a person’s opportunities for an experience of good mental health. This finding is in line with the current scientific literature on mental health risks, where some studies reported higher risks for mental disorders in people with mild or moderate ID [43, 44] while others reported higher risks in people with severe or profound ID [45]. This highly heterogeneous data basis might reflect on the disagreement upon our interview partners.

Although no difference was drawn between the mental health of people with and without ID, it was pointed out that the social context plays a more important role for people with ID. The interviewees responded highly concurrent when asked to identify the most important aspects of good mental health for people with ID: They prioritized self-determination and respect for people with ID. This includes treating them as adults and trying to see their resources, not only their deficits. Moreover, social participation and a benevolent, supportive environment were considered crucial for good mental health. These results correspond with the findings of van Herwaarden and colleagues [46], who reported that social acceptance, belonging and positive relationships are fundamental to the emotional wellbeing of people with ID. Similar conclusions may also be drawn for other marginalized groups, like people of color or the LGBTQIA + community. e.g [47–49].

### Limitations

In our sample, psychiatrists were slightly overrepresented (*n* = 5) and nurses slightly underrepresented (*n* = 3). Moreover, our participants identified predominantly as female (9 out of 12). As our findings are based on the personal experiences and opinions of twelve mental health experts, their views may not be representative of all professionals considered experts on good mental health in people with ID. Even though code saturation was reached in this study, another group of experts working with people with ID (e.g., occupational therapists, social workers, speech and language therapists) may consider some other factors to be important for good mental health in people with ID. However, to our knowledge, this is the first expert interview study examining the construct of good mental health in people with ID. Its exploratory nature represents a major strength and allows for the synthesis of expert opinions, providing a first comprehensive collection of factors contributing to good mental health of people with ID. Further research should aim to broaden the target groups in the exploration of this topic. This could include qualitative interviews or focus groups with other professions working with people with ID, close family, or professional caregivers as well as directly involving people with ID as study participants, but also as co-researchers in an inclusive research setting. The latter would require adapting research methods and collaboration strategies between academic researchers and co-researchers with ID, to make them accessible for all members of the research team.

## Conclusions

It can be concluded that mental health experts consider similar factors relevant for good mental health of people with and without ID. For people with ID, however, an environment that suits a person’s individual needs, as well as a benevolent social network and needs-based, person-centered support services are particularly relevant for this heterogenous population. This indicates the relevance of adapting the environment to suit the personal needs of people with ID, which is consistent with the social model of disability. The identification of the factors pertaining to good mental health in people with ID, could ultimately facilitate the development of targeted mental health promotion programs, which would represent a relevant contribution to the advancement of health equity for people with ID.

## Electronic supplementary material

Below is the link to the electronic supplementary material.

Supplementary Material 1

Supplementary Material 2

Supplementary Material 3

## Data Availability

Due to the nature of the research and to protect the privacy and anonymity of participants, data cannot be shared.
