# Correction: Exploring good mental health for people with intellectual disabilities: a qualitative interview study with mental health experts

**DOI:** 10.1186/s12939-025-02585-1

**Published:** 2025-07-16

**Authors:** Sophie Komenda-Schned, Paula Moritz, Sarah Jasmin Landskron, Alma Rosa Herscovici, Charlotte Schomburg, Julia Lehner, Brigitte Lueger-Schuster, Luis Salvador-Carulla, Elisabeth Lucia Zeilinger

**Affiliations:** 1 Department of Clinical and Health Psychology, Faculty of Psychology, University of Vienna, Liebiggasse 5, Vienna, A-1010 Austria; 2 Vienna Doctoral School in Cognition, Behavior and Neuroscience, University of Vienna, Vienna, Austria; 3 Research Institute for Developmental Medicine, Johannes Kepler University Linz, Linz, Austria; 4 Health Research Institute, Faculty of Health, University of Canberra, ACT, Canberra, Australia; 5 Department of Clinical Research SBG, Academy for Ageing Research, Haus der Barmherzigkeit, Vienna, Austria

**Correction: Int J Equity Health 24, 172 (2025).**

10.1186/s12939-025-02540-0

Following publication of the original article [1], it was reported that in the Materials and Methods section, four instances of anonymized text remaining from the peer review process were present.

In the following sentences, the bolded text has replaced ‘blinded for review’:

1. The interview questions were developed by two psychologists (**SKS**,** PM**) with scientific and practical experience in the field of mental health and ID.

2. Interviews were conducted by two trained members of the research team (**SKS**,** PM**).

3. Initially, a set of three interviews with representatives of different mental health professions, reporting a broad range of experts’ perspectives, was collectively coded by all members of the coding team (**PM**,** AH**,** JL**,** CS**) to develop a comprehensive and consistent coding scheme.

4. To avoid interpretation bias, two researchers who were not previously involved in the coding process (**SKS**,** SJL**) reviewed and adapted the thematic mapping.

The original article [1] has been corrected.
