# Supplementary material for: Exploring good mental health for people with intellectual disabilities: a qualitative interview study with mental health experts
Source: Int J Equity Health. 2025 Jun 12;24:172. doi: 10.1186/s12939-025-02540-0 (PMC12164140; doi:10.1186/s12939-025-02540-0)
Supplement: Supplementary file 1 — Supplementary Material 1 [file 12939_2025_2540_MOESM1_ESM.pdf]

Supplementary File A.

Interview Guidelines

Introductory questions

- At the beginning, could you briefly describe your professional environment?
- Why are you working in this specific field (with people with ID)?
    - Personal involvement?
    - Other experience?
    - Coincidence?
    - Career path?
- Practical experience with people with ID
    - In which way do you work with people with ID?
    - How regularly do you work with people with ID?

Mental Health in general

- What do you understand by mental health in general?
    - What are important factors of mental health?
- How can you tell that a person is mentally healthy?

Mental Health for people with ID

- What do you understand by mental health in relation to people with ID?

DEBRIEFING: mental **health** not illness

- What constitutes mental health in this population?
- How would you know that a person with ID is mentally healthy?
    - In your opinion, are there differences to the mental health of the general population?
    - Which factors of mental health are particularly relevant for people with ID?
- Are there differences in the definition of mental health in relation to people with **different levels of ID / different etiologies**?

- Are there differences in the definition of mental health in relation to people with different levels of **independence in everyday life** (in relation to living or working → **levels of functioning**, high/medium/low support needs)?
- Are there differences in the definition of mental health in different **life stages of adults** with ID?

Promoting mental health for people with ID

- What do people with ID need in order to stay mentally healthy?
  - Can you describe specific (case) examples/experiences in more detail regarding factors maintaining/promoting mental health?
- How can the mental health for people with ID be promoted?
  - What can people with ID do in order to promote their mental health?
  - What can support persons do?
  - What can professionals do?
- What **support services** do you think are needed for people with ID to promote mental health?
- What role do **societal or sociopolitical aspects** play with regard to the mental health of people with ID?

Mental health in people with ID - barriers

- What factors hinder mental health in people with ID?
  - What factors are promoting mental illness?
  - Can you describe specific cases/experiences regarding factors promoting/maintaining mental illness in more detail?

Closing

- What do you think are the **most important factors** contributing to mental health in people with ID?
- What do you think are the **top-3-factors** contributing to mental health in people with ID?
