# Supplementary material for: Exploring good mental health for people with intellectual disabilities: a qualitative interview study with mental health experts
Source: Int J Equity Health. 2025 Jun 12;24:172. doi: 10.1186/s12939-025-02540-0 (PMC12164140; doi:10.1186/s12939-025-02540-0)
Supplement: Supplementary file 2 — Supplementary Material 2 [file 12939_2025_2540_MOESM2_ESM.pdf]

Demographics of Contacted Mental Health Experts

name & contact details	profession	gender	work setting	level of experience	nationality
deleted to protect the anonymity of our participants	psychologist, psychotherapist	f	clinical	high	AT
	psychologist, psychotherapist	f	clinical	high	DE
	psychologist, psychotherapist	m	clinical	high	UK
	psychologist, psychotherapist	f	clinical	high	AT
	psychologist, psychotherapist	f	clinical	high	AT
	psychologist, psychotherapist	f	clinical	high	DE
	psychologist, psychotherapist	m	clinical/scientific	high	DE
	psychiatrist	m	clinical/scientific	high	AT
	psychiatrist	m	clinical	high	DE
	psychiatrist	m	clinical	high	AT
	psychiatrist	f	clinical	high	AT
	psychiatrist	f	clinical/scientific	high	NL
	psychiatrist	f	clinical/scientific	high	DE
	psychiatric nurse	f	clinical	high	AT
	psychiatric nurse	m	clinical/scientific	high	UK
	psychiatric nurse	f	clinical	high	DE
	psychiatric nurse	f	clinical	high	DE
	psychiatric nurse	f	clinical	high	DE
	psychiatric nurse	m	clinical	high	AT
	psychiatric nurse	f	clinical	high	DE
