# Supplementary material for: Exploring good mental health for people with intellectual disabilities: a qualitative interview study with mental health experts
Source: Int J Equity Health. 2025 Jun 12;24:172. doi: 10.1186/s12939-025-02540-0 (PMC12164140; doi:10.1186/s12939-025-02540-0)
Supplement: Supplementary file 3 — Supplementary Material 3 [file 12939_2025_2540_MOESM3_ESM.pdf]

Supplementary File C.

Contextual Theme: Ways of Mental Health Promotion

Ways of Mental Health Promotion was included as a contextual theme, since it was not our primary research interest. It was, however, mentioned by many interviewees and therefore added as a secondary finding. This contextual theme was divided into four subthemes: (1) Interdisciplinary work, (2) ID-specific Knowledge, (3) Specialized Offers for People with ID, and (4) Available Resources.

Interdisciplinary Work

Cooperation between the health and the social sector is one possibility for promoting mental health in people with ID, as pointed out in several interviews.

> *So, all this professionalized, interdisciplinary work, that not everybody is always simmering in their own area. I think, that´s very important.* (Interview 4)

Good communication within the support system as well as with the people with ID themselves, is crucial to prevent information loss and enables the best support possible. However, the current cooperation has been described as follows:

> *What I clearly see is that there are two worlds, so to speak. The disabled world and the psychiatric world. They are only connected to a certain extent by chance via individual hinges. If someone says 'I used to work in a psychiatric ward, but for the last five years I´ve been in an institution for people with disabilities' or vice versa, that is obviously not enough. […] This must be systematically organized.* (Interview 5)

ID-specific Knowledge

To ensure mental health in people with ID in the long term, specific training and further education for care persons as well as members of the professional health care system on (mental) health for people with ID is necessary, regarding to our interview partners.

> *We need to anchor the topic of disability and the specifics of disability much more firmly in the training and further education of all professional groups working in the health care sector.* (Interview 5)

This includes basic training on disability-specific content as regular part of the studies or apprenticeship, as well as the possibility to get a post-gradual specialization in the (mental) health care work with people with ID.

> *That it is a matter of course to have this [specialization] in the whole canon of different specializations. Or that it is an integral part in the training of psychotherapists, as one [patient] group among many. You can´t say, 'Oh, I´m not interested in depression, I´m not going there now.' So that it is simply a matter of course.* (Interview 4)

However, it is important to point out both, the differences as well as the similarities between people with and without ID.

> *The professional health care system is generally not trained to see people with intellectual disabilities in their specialty and at the same time in their commonality with the average population.* (Interview 11)

Specialized Offers for People with ID

In some cases, medical facilities for the general population are not suitable for people with ID because of limitations in accessibility, too little knowledge about the (medical) needs of people with ID, or organizational matters like crowded waiting areas and long waiting times. Moreover, specialized psychosocial services (e.g., psychotherapy, consulting, psychoeducation) are urgently needed for this population, as mentioned in many interviews.

> *The first starting point is, of course, the promotion of health literacy concerning mental health.* […] *We need psychoeducation and empowerment regarding mental health with methods and didactics adapted to the target groups.* (Interview 5)

Apart from medical and psychosocial offers information on specific recreational activities for people with ID should be organized centrally to make it easily available for everybody.

> *I think an information hub would be quite cool. If there was an information hub somewhere that simply collected information and passed it on. Information on how I can organize leisure activities well. [...] And it can range from A like riding an alpaca or walking with an alpaca to Z for petting a zebra. Where can you do that? Who is the contact person? And I think that would be really cool.* (Interview 7)

Available Resources

Sufficient personal and material resources are needed in the medical sector as well as in supported living or working facilities to provide individual, needs-oriented assistance. The interviewees pointed out various aspects related to this matter.

> *And there is the time factor, i.e. that there is more time in the health care sector to build relationships.* (Interview 9)

> *This also requires well shaped accompanying personalities. Well-shaped in the sense that you have an instinct on one hand, but also learn how to interpret the person´s impulses and acquire technical skills. In this sense, good supporters are artists.* (Interview 11)
